# Gene mutation profiling and clinical significances in patients with renal cell carcinoma

**DOI:** 10.1016/j.clinsp.2023.100259

**Published:** 2023-07-27

**Authors:** Yongquan Wang, Peng He, Xiaozhou Zhou, Cong Wang, Jian Fu, Dawei Zhang, Deyang Liao, Zhansong Zhou, Chunman Wu, Wei Gong

**Affiliations:** a Department of Urology, Southwest Hospital, Third Military Medical University (Army Medical University), Shapingba District, Chongqing, China; b Department of Biochemistry, Third Military Medical University (Army Medical University), No. 30 Gaotanyan Road, Shapingba District, Chongqing, China; c Medicine Department, Nanjing Geneseeq Technology Inc, Nanjing, Jiangsu, China

**Keywords:** BAP1, Next generation sequences, Overall survival, PBRM1, Renal cell carcinoma, Renal score

## Abstract

**Objectives:**

The pathological mechanisms of patients with Renal Cell Carcinoma (RCC) remain defined. This study aimed to evaluate relationships between the landscape of gene mutations and their clinical significance in RCC patients.

**Methods:**

Tissue and peripheral blood samples of 42 patients with RCC were collected and performed for the Next Generation Sequencing (NGS) with Geneseeq PrimeTM 425-gene panel probes. Their landscapes of gene mutation were analyzed. We also carried out an evaluation of Tumor-Node-Metastasis (TNM) staging, RENAL nephelometry score, surgery, and targeted drug treatment of patients. Then we compared the correlations of landscape in gene mutations and the prognosis.

**Results:**

The most common gene alternations, including BAP1, PBRM1, SETD2, CSF1R, NPM1, EGFR, POLE, RB1, and VHL genes, were identified in tissue and blood samples of 75% of patients. EGFR, POLE, and RB1 gene mutations frequently occurred in relapsed and metastatic patients. BAP1, CCND2, KRAS, PTPN11, ERBB2/3, JAK2, and POLE were presented in the patients with > 9 RENAL nephelometry score. Univariable analysis indicated that SETD2, BAP1, and PBRM1 genes were key factors for Disease-Free Survival (DFS). Multivariable analysis confirmed that mutated SETD1, NPM1, and CSF1R were critical factors for the Progression Free Survival (PFS) of RCC patients with target therapy.

**Conclusions:**

Wild-type PBRM1 and mutated BAP1 in patients with RCC were strongly associated with the outcomes of the patient. The PFS of the patients with SETD2, NPM1, and CSF1R mutations were significantly shorter than those patients without variants.

## Highlights

• EGFR, POLE, and PB1 genes had high mutated frequency in the patients with relapse and metastatic cancer.
• PBRM1, BAP1, KDM5C, and BAP1 genes were critical for the overall survival.
• Wild-type PBRM1 and mutated BAP1 were key indications for the predication of OS.

## Introduction

Globally, Renal Cell Carcinoma (RCC) is the most common type of kidney cancer, which accounts for 90%‒95% of all cases.^1^ Incidence of RCC in men is significantly higher than that in women. RCC is originally from the malignant transformation of epithelial cells in the proximal convoluted tubule of the kidney. The treatments of RCC include surgery,^2^ targeted therapy,^3^ and immunotherapy,^4^ etc. The outcomes of patients with RCC have obtained great improvements in the past decades because of these new therapies. The risk factors for RCC development include smoking, obesity, hypertension, and genetics.^5^^,^^6^ Like other cancers, RCC patients harbor many gene mutations including K-ras, BIRC5, XIAP, MCL-1, HIF1 alpha, HIF2 alpha, and AKT.^7^ Especially, the mutation of Von Hippel-Lindau (VHL) gene in chromosome 3 is frequently associated with RCC.^8^ VHL gene mutation caused the initial occurrence of the tumor, then real carcinoma progressed with the loss of large fragment in the chromosome 3p region. The most common pathological subtype of RCC is clear cell Renal Cell Carcinoma (ccRCC), which accounts for nearly 85% of metastatic (mRCC).^8^

The VHL gene was originally cloned from a patient with VHL disease in 1993.^9^ The function of the VHL gene is as a tumor suppressor gene, which plays critical roles in regulating cell proliferation, differentiation, and apoptosis.^10^ Interestingly, it was found that at least one allele of the VHL gene in more than 90% of ccRCC patients was lost.^11^ In addition to VHL gene mutation, SETD2, BAP1, MTOR, PTEN, KDM5C, and PBRM1 gene mutations were also frequently identified in chromosome 3p regions of most ccRCC patients.^12^ These findings indicated that VHL mutation is not only a critical biomarker of ccRCC but also a key factor for the pathogenesis of ccRCC. Therefore, VHL gene mutation became a major target for the therapy of ccRCC. Indeed, since the VHL gene mutation was cloned in ccRCC patients, a few key drugs that targeted the VHL gene have developed.^13^, ^14^, ^15^, ^16^ For example, HIF-2α inhibitor PT2385 targeting VHL gene mutation has obtained 66% overall Disease Control Rate (DCR) in 51 RCC patients.^17^ In addition, belzutifan treatment for RCC patients has achieved 91.8% (56/61) tumor size reduction. It was also shown that this drug can treat Central Nervous System (CNS) hemangioblastoma, retinal hemangioblastoma, and pNET disease.^14^^,^^18^

Although these great accomplishments have been achieved, the other gene mutations and the prognosis significances of gene mutations in mRCC patients remain defined. Here, we collected tumor tissues and peripheral blood samples from 42 patients with renal cell carcinoma and performed Next-Generation Sequencing (NGS) for these patients, which can detect a large number of gene mutations in a short time.^19^^,^^20^ In the past decades, NGS was widely used to apply diagnosis, treatment, and drug resistance to disease.^21^^,^^22^ This study aimed to monitor gene mutation profiling and evaluate relationships between landscapes of gene mutation and the prognosis significances of renal cancer patients.

## Materials and methods

### Patients and samples

A total of 42 tumor tissues and peripheral blood samples were collected from urinary system diseases after biopsy, including 26 ccRCCs, 4 Eosinophilic Variant Clear Cell Carcinomas (evRCC), 4 papillary Renal Cell Carcinomas (pRCC), 4 chromophobe Renal Cell Carcinomas (chRCC), and 4 Mit family translocations RCC for gene mutation analysis in our department from January 2018 to April 2021. The criteria of enrolled patients were the following: (i) Patient's age was > 18 years old; (ii) No treatments before tumor and blood samples collection; (iii) Pathological subtypes were based on World Health Organization (WHO) recommended criteria;^23^ (iv) The stages of RCC patients were following the 8^th^ edition of AJCC.^24^ At the same time, we also isolated DNA from the peripheral blood of patients as a control. The clinical characteristics of 42 patients were shown in Table 1. This study protocol was reviewed and approved by the Ethics Committee of the affiliated Southwest Hospital of Army Medical University (Approval nº KY2020121). Written informed consent has obtained from all participants before the study.

**Table 1:** The clinical features of 42 patients with renal clear cell cancer.

	Number of Cases	χ2	p-values
Age		1.32	0.87
> 60-year-old	20 (47.6%)		
≤ 60-year-old	22 (52.4%)		
Gender		0.04	0.02
Female	14 (33.3%)		
Male	28 (66.7%)		
Pathological subtype		12.53	0.001
ccRCC	26 (61.9%)		
pRCC	4 (9.5%)		
chRCC	4 (9.5%)		
evRCC	4 (9.5%)		
RCC with Mit family translocation	4 (9.5%)		
Renal Score		10.25	0.001
> 9	15 (35.7%)		
≤ 9	27 (64.3%)		
Surgery		13.25	0.002
Yes	30 (71.4%)		
No	12 (28.6%)		
Targeted drug use		1.54	0.97
Ye	21 (50%)		
No	21 (50%)		

ccRCC, Clear Cell Renal Cell Carcinoma; evRCC, Eosinophilic Variant Clear Cell Carcinoma, pRCC, Papillary Renal Cell Carcinoma, chRCC, Chromophobe Clear Cell Carcinoma.

### DNA preparation and the next generation sequence (NGS)

DNA isolation from tumor and peripheral blood samples was described in as previous publication.^25^ Briefly, Formalin-Fixed and Paraffin-Embedded (FFPE) tumor samples before treatment were collected and shipped to the core facility of Nanjing Shihe Jiyin Biotechnology Inc (Nanjing, China) for gene mutation analysis. Around 5 to 10 milliliters (mL) of peripheral blood was drawn from the patient and transferred into EDTA-coated tubes (BD Biosciences), then peripheral blood was loaded into 50 mL tube with Ficoll-Paque Plus solution (GE, USA) and spin 30 minutes at 961g in Eppendorf Centrifuge 5810R with A-4-62 swing bucket rotor. Taking a middle white layer cell and processing for DNA isolation. Genomic DNA preparation was performed with DNeasy Blood & Tissue kit (QIAGEN). The DNA quality was assessed by Nanodrop2000 (Thermo Fisher Scientific), and the quantity was measured by dsDNA HS Assay Kit (Life Technologies) on Qubit 2.0.

### Targeted NGS and gene mutation analysis

After the above DNA extraction from tumor tissue and peripheral blood samples, sequencing libraries were constructed using the KAPA Hyper Prep Kit (KAPA Biosystems) following to manufacturer's instructions and were hybridized with probes targeting 425 cancer-relevant gene probes (Geneseeq Technology Inc). These probes can specifically bind 425 key biomarkers, including Tumor Mutation Burden (TMB) and Microsatellite Instability (MSI).^26^ The capture reactive conditions were carried out with Dynabeads M-270 (Life Technologies) and xGen Lockdown hybridization. The library quantification assessment by qPCR was performed with the KAPA Library Quantification kit (KAPA Biosystems). All interested fragment were sequenced on the HiSeq4000 NGS platform (Illumina, USA).

### Sequence alignment and processing

Read bases were aligned via bcl2fastq v2.16.0.10 software (Illumina, Inc.) to generate sequence results in FASTQ format (Illumina 1.8+ encoding). Huge base-pairs were aligned to the human genome (hg19, GRCh37 Genome Reference Consortium Human Reference 37) using the BWA aligner 0.7.12 with BWA-MEM algorithm and default parameters to create SAM files, which convert to compressed BAM files.

### SNVs / Indels / CNVs detections

Single Nucleotide Variants (SNVs) and short insertions/deletions (indels) mutants were confirmed by VarScan2 2.3.9 with minimum variant allele frequency threshold set at 0.01, and p-value threshold for calling variants set at 0.05 to generate Variant Call Format (VCF) files. Each SNV/indel was manually checked on the Integrative Genomics Viewer (IGV). Protein and amino acid sequence changes caused by gene mutations were checked using ANNOVAR software,^27^ Copy Number Variations (CNVs) were detected via SIFT^28^ and PolyPhen^29^ software.

### Statistical analysis

Categorical variables were presented as percentage and frequencies. Chi-Square test was used to compare categorical variables. For Progression Free Survival (PFS) and Disease-Free Survival (DFS) analysis, Kaplan-Meier curves were used via a log-rank test. Statistical analysis was performed with GraphPad Prism software, version 9.0 (GraphPad software Inc) and R software, version 3.5.0 (R Foundation for Statistical Computing). The cutoff of p-values (< 0.05) was considered as a significant difference.

## Results

### Clinical characteristics

To investigate relationships between gene mutations and the outcomes of RCC patient, we recruited 42 patients with renal cell carcinoma as our study objective. Their clinical characteristics show in Table 1. These patients included 14 female (33.3%) and 28 male patients (66.7%). The number of female patients were significant less than that of male patients (p < 0.05). The number of patient with > 60 year-old 20 (47.6%) was almost equal to patients with ≤ 60 year-old 22 (52.4%). The patients were divided into ccRCC 26 (61.9%), eosinophilic variant clear cell carcinoma (evRCC,4, 9.5%, papillary renal cell carcinoma (pRCC,4, 9.5%), chromophobe RCC (chRCC,4, 9.5%), and RCC with Mit family translocation (4, 9.5%) according to H&E staining and cell morphology, respectively. The ccRCC patients were dramatically higher than other pathological subtypes (p = 0.001). The patient number with ≤ 9 renal score 27 (64.3%) was dramatically more than patient number with > 9 renal score 15 (35.7%) (p = 0.001). Among 42 patients, 30 cases (71.4%) were performed surgery and 21 cases (50%) were carried out targeted drug treatment.

### Gene mutation landscape of ccRCC by NGS

**Fig. 1:**
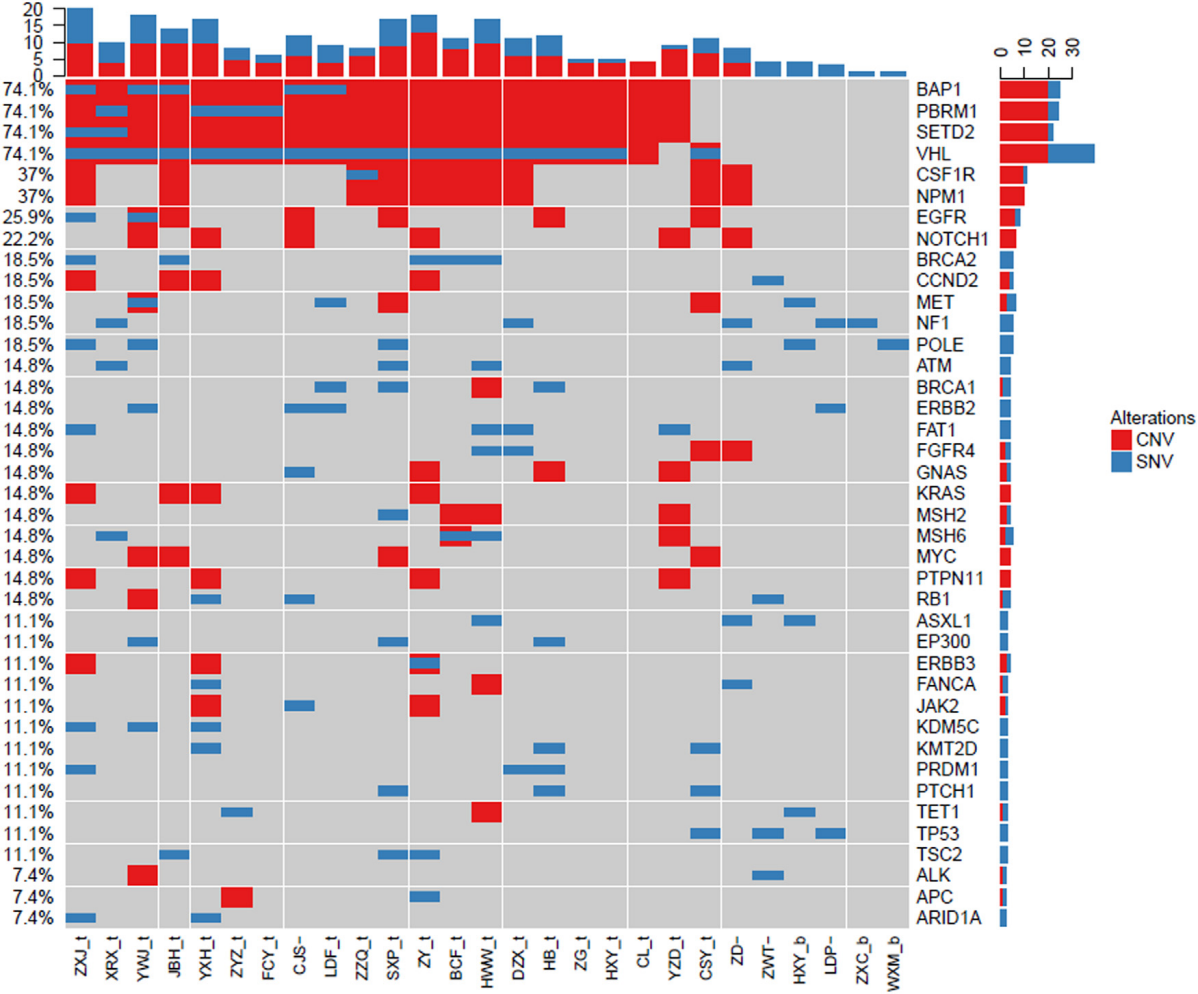
Landscape of gene mutations from patients with clear cell renal cell carcinoma (ccRCC): Left Y-axis shows frequent mutated genes identified by MutSig CV and Lauren classification. Right Y-axis shows gene names. X-axis shows abbreviation of patient name. t, tumor; b, blood; CNV, Copy Number Variation; SNV, Single Nucleotide Variation.

To investigate the landscape of gene mutations in 26 patients with ccRCC, we collected the tumor tissues from biopsy and peripheral blood from patients before treatment and carried out NGS test. The results are shown in Fig. 1. The most common mutated genes were BAP1 (74.1%), PBRM1 (74.1%), SETD2 (74.1%), and VHL (74.1%), which are slightly higher that data in the Cancer Genome Atlas (TCGA).^30^ Except those frequent mutated genes in urinary system cancers, we also identified other less frequent mutated genes, including CSF1R (37%), NPM1 (37%), EGFR (25.9%), and NOTCH1 (22.2%). Each gene mutation had different alternation styles, which hold differential CNV and SNV ratios. BAP1, PBRM1, SETD2, CSF1R, and NPM1 mainly had Copy Number Variation (CNV) mutations. In contrast, VHL gene mutation was mixed with CNV and Single Nucleotide Variants (SNV). Interestingly, we found that CSF1R, NPM1, and EGFR in our top 50 mutations list didn't show in TCGA database. These results indicated that CSF1R, NPM1, and EGFR gene mutations may be also involved in the pathogenesis of ccRCC except frequent BAP1, PBRM1, SETD, and VHL gene mutations.

### Gene mutation style of the other subtype RCC

**Fig. 2:**
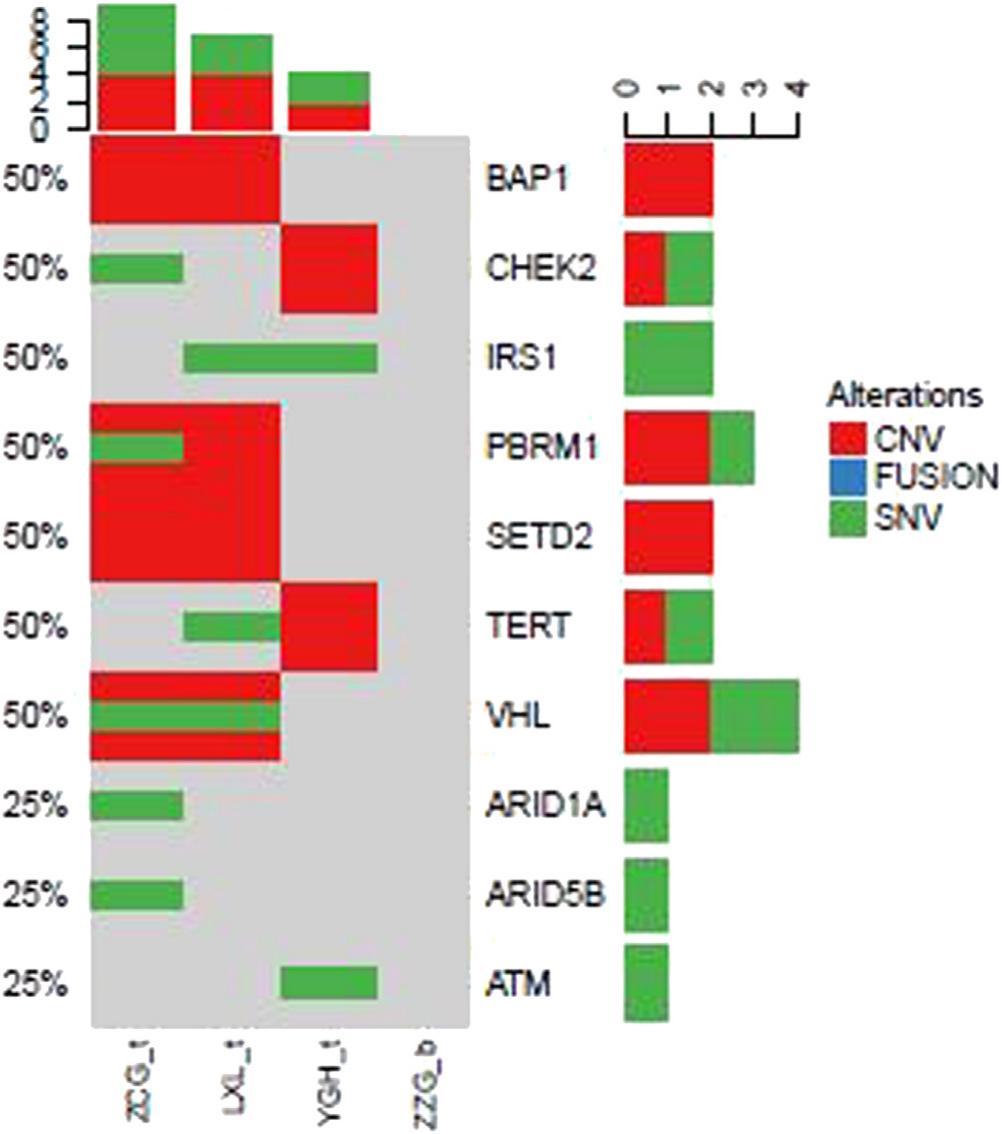
Landscape of mutations from patients with clear cell renal cell carcinoma (ccRCC) subtype: Left Y-axis shows frequent mutated genes identified by MutSig CV and Lauren classification. Right Y-axis shows gene names. X-axis shows abbreviation of patient name. t, tumor; b, blood; CNV, Copy Number Variation; SNV, Single Nucleotide Variation.

In addition to above gene mutations landscape of ccRCC patients, we also identified top 10 gene mutations profiles of other subtype RCC. Compared to ccRCC (Fig. 2) BAP1, CHEK2, IRS1, PBRM1, SETD2, TERT, and VHL in evRCC patients, PAX8, CDKN2A, CDKN2B, FANCC, FGFR4, PIK3C, PTPRS, SMARCB1, TERT in pRCC ASXL2, BRCA1, CDH1, CDKN2A, CDKN2B, CDKN2C, DNMT1, and EP300 mutations in chRCC patients were mainly found. This result indicated that different subtype RCC had its own mutation style. Especially, there were distinctive differences between evRCC and pRCC or chRCC. These differentiated gene mutation profiles may play critical role in the occurrence of different subtype RCC. Of course, limited sample sized may cause bias of gene mutations landscape.

### Gene mutation profiles of in different clinical feature groups

**Fig. 3:**
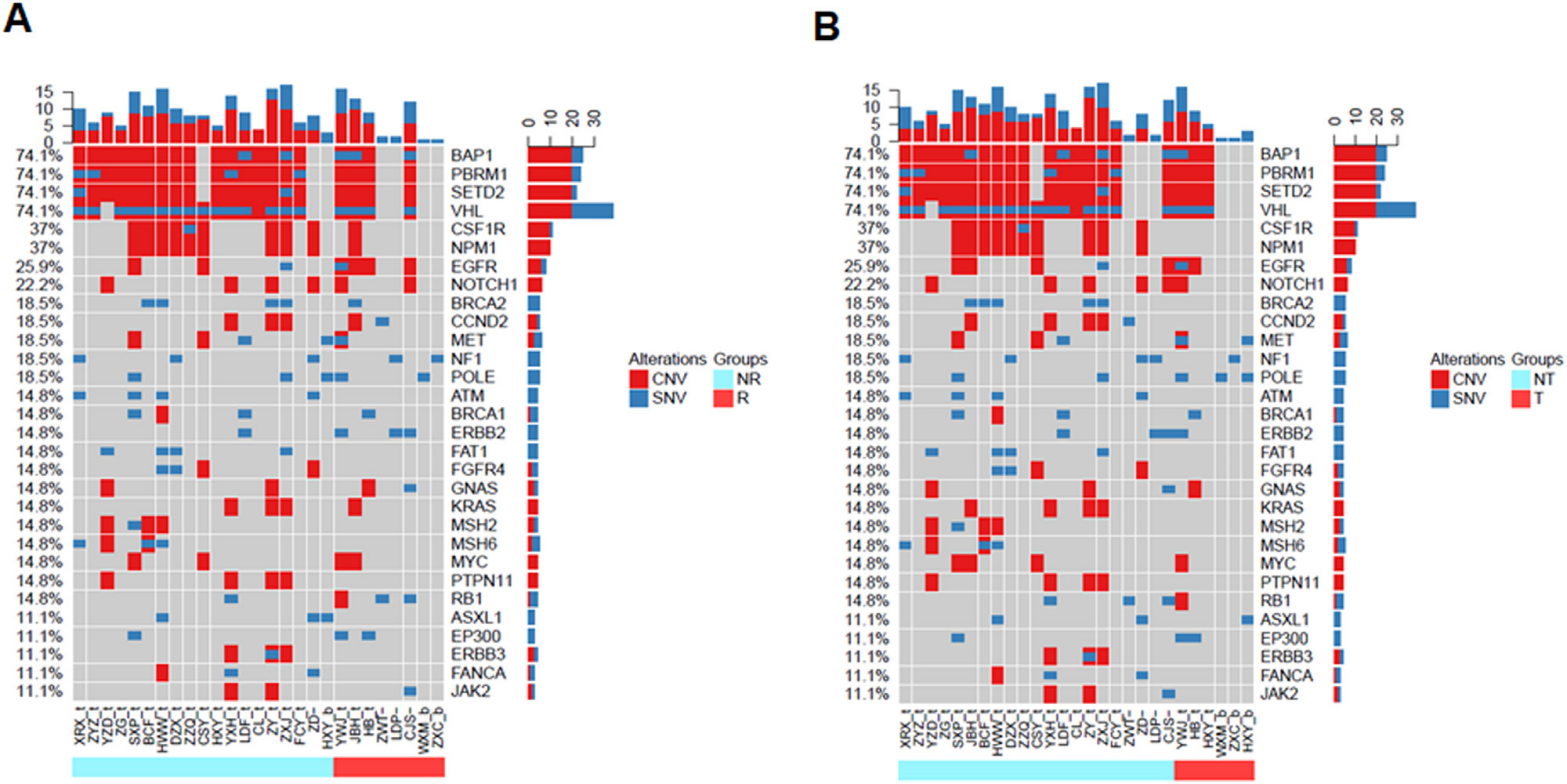
Landscape of mutations from RCC patients with and without recurrence or metastasis. (A) Landscapes of gene mutations from recurrence and non-recurrence. (B) Gene mutations frequently occurred in metastasis (transfer) and non-metastasis (transfer) patients. Left Y-axis shows frequent mutated genes identified by MutSig CV and Lauren classification. Right Y-axis shows gene names. Top x-axis shows abbreviation of patient name. Bottom X-axis shows that either non-recurrence and non-transfer (light blue color) groups or recurrence and transfer (red color) groups. t, tumor; b, blood; CNV, Copy Number Variation; SNV, Single Nucleotide Variation; NR, Non-Recurrence; R, Recurrence; T, Transfer; NT, Non-Transfer.

To validate the prognostic impacts of different clinical feature groups, we compared the gene mutation landscapes of the patients with and without recurrence or metastasis (Fig. 3). We found that there were BAP1, PBRM1, SETD2, VHL1, CSF1R, NPM1, BRCA2, CCND2, ATM, FAT1, FGFR4, KRAS, MSH2, MSH6, PTPN11, and ERBB3 in non-relapse (Fig. 3A, light blue group) or without metastatic patients (Fig. 3B, light blue group). In contrast, EGFR, POLE, and RB1 gene mutations frequently occurred in recurrence (Fig. 3A, red color group) and metastatic patients (Fig. 3 B, red color group).

**Fig. 4:**
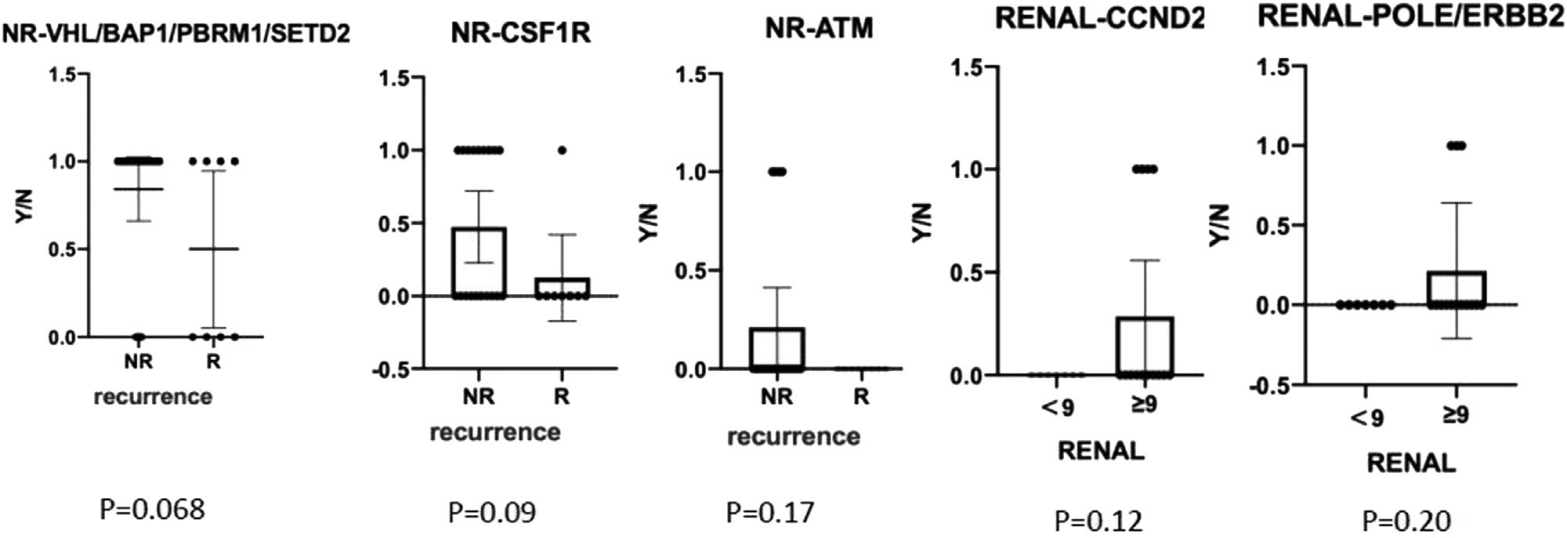
Gene mutation comparison of RCC patients with Recurrence (R) or non-recurrence (NR). This graph was comparison of chance non-recurrence and recurrence patients with different gene mutations.

We also compared gene mutation profiles in patients with or without recurrences and different RENAL Nephrometry Score (Fig. 4). The results indicated that BAP1/PBRM1/SETD2/CSF1R/ATM mutations in non-recurrence patients were higher than that in recurrence patents. CCND2, ERBB2, and POLE gene mutations usually existed in RENAL Score > 9. In contrast, there were frequently FAT1, MSH2/6, ATM, and FGFR4 gene mutations in RENAL Score < 9. PBRM1, CSF1R, NPM1, MSH2/6, CCND2, FAT1, KRAS, PTPN11, and TSC2 gene mutations frequently occurred in patient with TNM stage I. In contrast, EGFR, POLE, and ERBB2 existed in patients with stage IV.

### The early disease-free survival (DFS) evaluation of the patients in different gene mutations

**Fig. 5:**
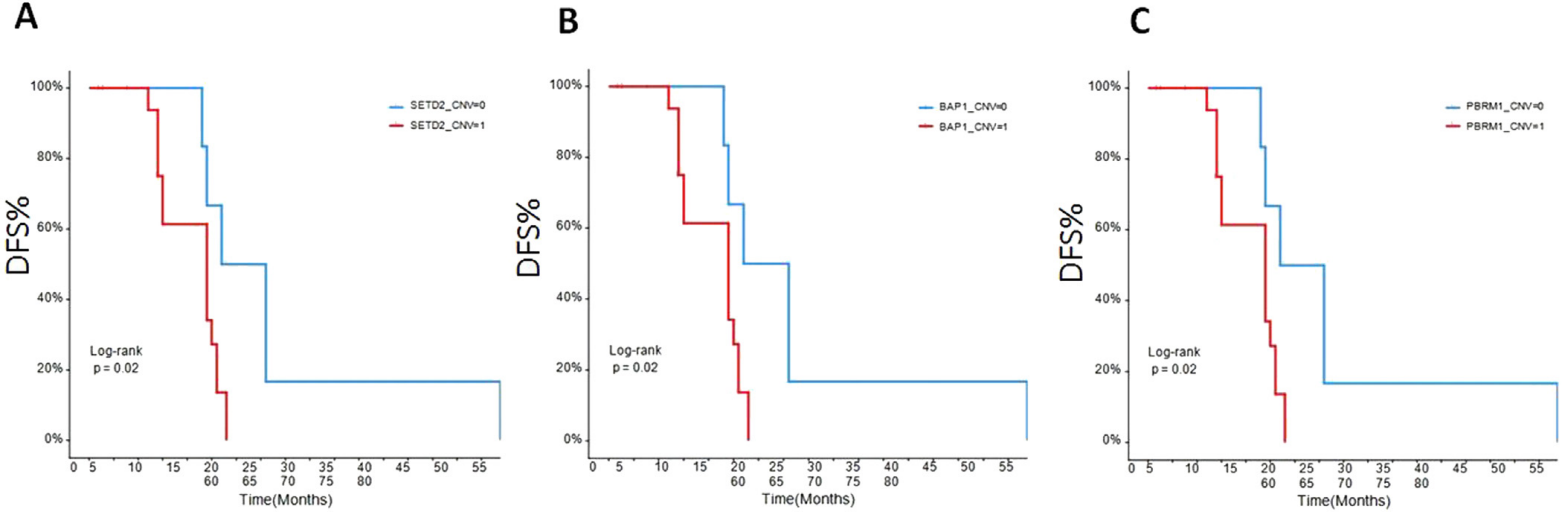
Comparison of disease-free survival in RCC patients with different gene mutations. (A) SETD2; (B) BAP1; (C) PBRM1. CNV-0, no copy number variation; CNV-1, with copy number variation.

**Fig 6:**
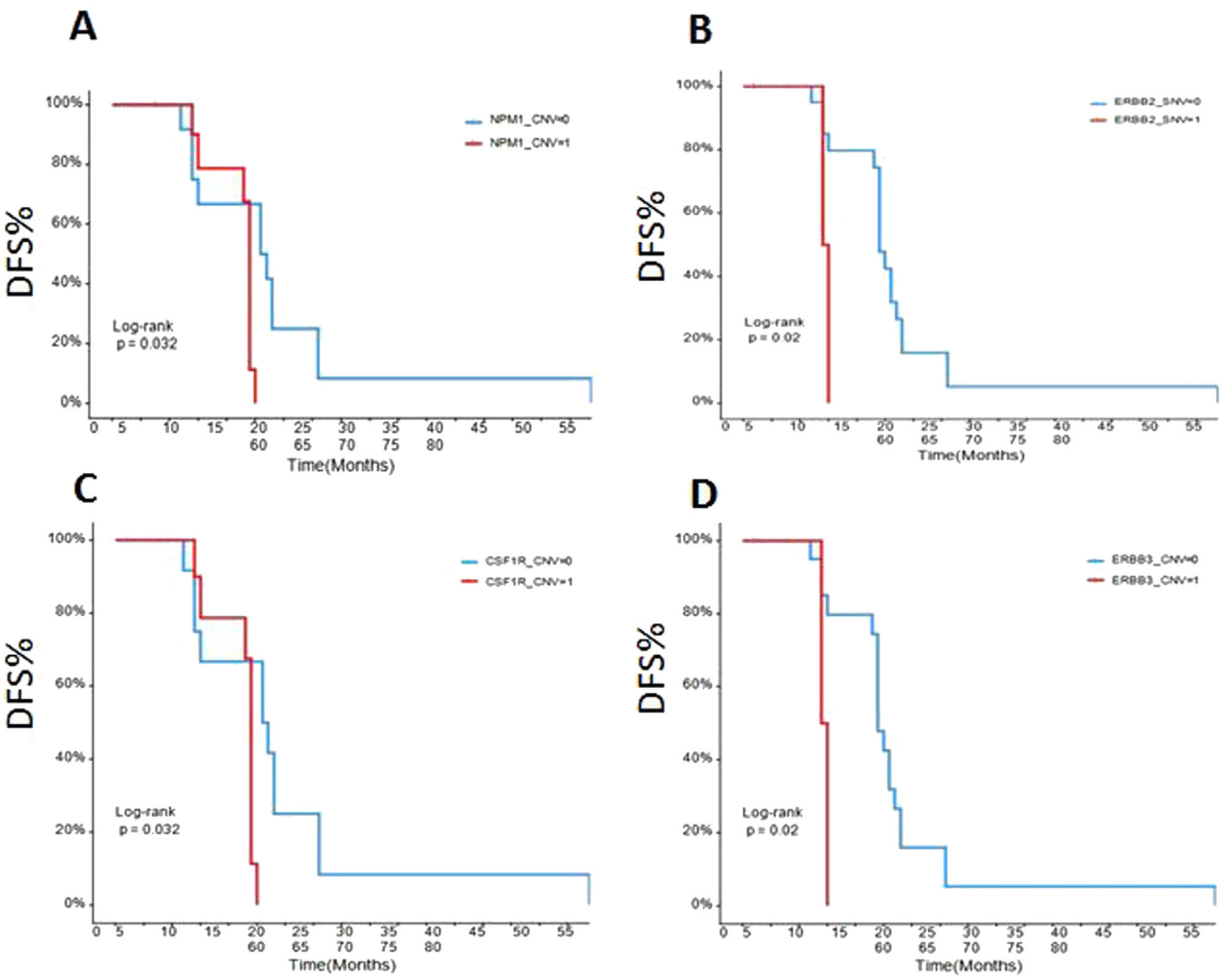
Comparison of disease-free survival in RCC patients with NPM1, CSFR1, ERBB2/3. (A) NPM1; (B) ERBB2; (C) CSFR1;(D) ERBB3. CNV-0, no copy number variation; CNV-1, with copy number variation.

To assess the relationships between mRCC patients with different gene mutations and DFS, we followed up the outcomes of 26 recurrence patients. The data showed that the DFS of the ccRCC patients with SETD2 (Fig. 5A), BAP1 (Fig. 5B), PBRM1 (Fig. 5C), NPM1 (Fig. 6A), ERBB2 (Fig. 6B), CSFR1 (Fig. 6C), and ERBB3 (Fig. 6D) gene mutations were significant shorter than that of wild type patients over time. This result indicated that SETD2/BAP1/PBRM1/NPM1/CSFR1, and ERBB2/3 gene mutations dramatically affect the progression of RCC patients.

### Characterization of gene mutations with PFS of targeted therapy

**Fig. 7:**
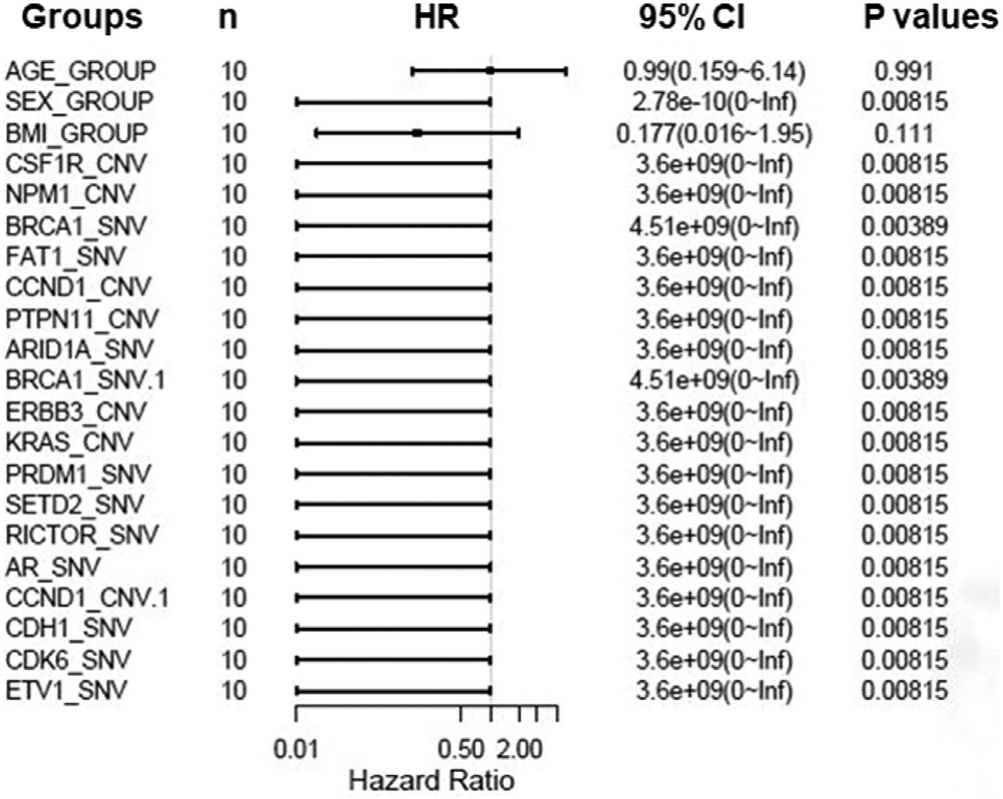
Hazard Ratios in 10 RCC patients with target therapy. HR, Hazard Ratio; CI, Confidence Interval; CNV, Copy Number Variant; SNV, Single Nucleotide Variant.

To further assess critical factors with prognosis of patients with targeted therapy, we reviewed PFS of 10 patients with target therapy and analyzed their gene mutant styles (Table 2). We found that patients with male, SETD2-_SNV, NPM1_CNV, and CSF1R_CNV mutations had significant poorer PFS than patients with wild type. In addition, multiple factors analysis also showed that BRCA1, FAT1, CCND1, PTPN11, and ARID1A gene mutations are key genes to determine prognosis in RCC patients (Fig. 7). All together, these data confirmed that NPM1, SETD2, CSF1R, and ERBB2/3 are important gene in predicting the prognosis of RCC patients.

**Table 2:** Progression free survival (PFS) relevant genes log-rank test values.

Gene names	HR (95% CI for HR)	Log-rank_ p-value
SEX_ group	0 (0-Inf)	0.008
CSF1R_CNV	3598241712.414 (0-Inf)	0.008
NPM1_CNV	3598241712.414 (0-Inf)	0.008
BRCA2_SNV	4262925654.7 (0-Inf)	0.008
FAT1_SNV	3598241712.414 (0-Inf)	0.008
CCND2_CNV	3598241712.414 (0-Inf)	0.008
PTPN11_CNV	3598241712.414 (0-Inf)	0.008
ARID1A_SNV	3598241712.414 (0-Inf)	0.008
ERBB3_CNV	3598241712.414 (0-Inf)	0.008
KRAS_CNV	3598241712.414 (0-Inf)	0.008
PRDM1_SNV	3598241712.414 (0-Inf)	0.008
SETD2_SNV	3598241712.414 (0-Inf)	0.008
RICTOR_SNV	3598241712.414 (0-Inf)	0.008
AR_SNV	3598241712.414 (0-Inf)	0.008
CDH1_SNV	3598241712.414 (0-Inf)	0.008
CDK6_SNV	3598241712.414 (0-Inf)	0.008
ETV1_SNV	3598241712.414 (0-Inf)	0.008

CNV, Copy Number Variations; SNV, Single Nucleotide Variants; HR, Hazard Ratio; CI, Confidence Interval.

## Discussion

Here, we performed NGS for the detection of individual gene mutation from 42 RCC patients. Our results showed that the most frequent mutations included BAP1 (74.1%), PBRM1 (74.1%), SETD2 (74.1%), and VHL (74.91%), respectively. These findings are consistent with previous reports.^8^^,^^12^ Interestingly, in addition to these common mutations in ccRCC patients, we also found some unreported gene mutations like CSF1R, NPM1, and EGFR in TCGA data base. Different subtypes of RCC patients had distinctive gene mutation profiles. SETD2, BAP1, PBRM1, NPM1, CSFR-1, and ERBB2/3 genes were critical factors to determine the outcomes and response to targeted therapy in RCC patients.

In this study, we identified 50 frequently mutated genes in 42 RCC patients. Like other reports, BAP1, PBRM1, SETD2, and VHL are always the most frequently mutated genes.^31^, ^32^, ^33^ Among these gene mutations, VHL play a critical role in ccRCC because at least one allele loss of VHL gene was identified in over 90% ccRCC patients.^8^^,^^11^^,^^34^ VHL gene is located in chromosome 3p short arm^34^ and loss of 3p usually occurs first through chromothripis, with VHL inactivation as a second event duo to the hypermethylation of the VHL promoter region.^32^ The loss of 3p as the first event typically occurs 5‒20 years before tumor diagnosis. PBRM1, BAP1, and SETD2, which are commonly observed in other mutated genes in sporadic ccRCC are coincidentally located on chromosome 3p. This confers the probability that the inactivation of PBRM1, BAP1, or SETD2 can also occur during the tumorgenesis of ccRCC, similar to VHL. Consequently, although VHL is the main player in the pathological biology of ccRCC, these other tumor suppressor clusters are also likely to be involved. In fact, recent studies have shown that VHL inactivation alone is not sufficient for the development of ccRCC. As previous descriptions, VHL is not only tumor suppressor genes, but also play other functions.^33^

Here, we also observed that BAP1 gene mutation plus wild type PBRM1 play a critical role in predicting the outcomes of RCC. Several studies investigated their prognostic values since 2014.^35^, ^36^, ^37^, ^38^ Some authors confirmed PBRM1 as an independent predictor PFS, but not Overall Survival (OS).^39^ The prognostic value of BAP1 was dependent on the cellular localization^40^ and need combine with the expression of PBRM1. Our results support this hypothesis. In addition, we analyzed that gene alteration style in patients with targeted therapy and found SETD2, NPM1, CSF1R, BRCA1, FAT1, CCND1, PTPN11, and ARID1A gene mutations are key genes to determine prognosis in RCC patients .Thiesen et al. reported that up to 30% ccRCC patients had CSF1R gene mutations.^41^ This data was a little low our finding. The other study showed EGFR and SGLT1 in RCC patients have high expression,^42^ but no EGFR gene mutation is available. Here, our data showed EGFR gene mutation is up to 25.9%, which clue EGFR is heavily involved in tumor genesis of RCC patients.

The present findings are very interesting for clinical of RCC patients. However, this study has some limitations: (i) The present data is cohort study from 42 patients. This sample size is limit; (ii) Gene mutations in RCC patients are how to involve in tumorigenesis of patients; (iii) Gene mutations of RCC patients may be relevant to the efficiency of drug treatment. These fields will be explored in the future.

## Conclusions

Taken together, we performed a comprehensive mutational landscape of 42 RCC patients. We showed that BAP1 gene mutation plus wild type PBRM1 had significant contributions to renal cancer PFS and DFS. This provided a theoretical foundation for targeting BAP1 gene and PBRM1 therapy.

## Ethical approval statement

This study protocol was reviewed and approved by the ethical committee of the affiliated Southwest Hospital of Army Medical University (KY202021). Written informed consent has obtained from all participants before study.

## Data availability statement

Data are available from the corresponding author on request.

## CRediT authorship contribution statement

**Yongquan Wang:** Conceptualization, Investigation, Data curation, Supervision, Validation, Writing – review & editing, Writing – original draft. **Peng He:** Investigation, Data curation, Supervision, Writing – review & editing, Writing – original draft. **Xiaozhou Zhou:** Investigation, Writing – review & editing, Writing – original draft. **Cong Wang:** Data curation, Validation, Writing – review & editing, Writing – original draft. **Jian Fu:** Data curation, Validation, Writing – review & editing, Writing – original draft. **Dawei Zhang:** Data curation, Validation, Writing – review & editing, Writing – original draft. **Deyang Liao:** Project administration, Writing – review & editing, Writing – original draft. **Zhansong Zhou:** Conceptualization, Data curation, Supervision, Writing – review & editing, Validation, Writing – original draft. **Chunman Wu:** Data curation, Supervision, Validation, Writing – review & editing, Writing – original draft. **Wei Gong:** Conceptualization, Data curation, Supervision, Validation, Writing – review & editing, Writing – original draft.

## Declaration of Competing Interest

The authors declare that they have no known competing financial interests or personal relationships that could have appeared to influence the work reported in this paper.

## References

[1] Rini B.I., Rathmell W.K., Godley P. (2008). Renal cell carcinoma. Curr Opin Oncol.

[2] Herr HW. (1999). Partial nephrectomy for unilateral renal carcinoma and a normal contralateral kidney: 10-year followup. J Urol.

[3] Quinn D.I., Lara P.N. (2015). Renal-cell cancer–targeting an immune checkpoint or multiple kinases. N Engl J Med.

[4] Martini A., Fallara G., Pellegrino F., Cirulli G.O., Larcher A., Necchi A. (2021). Neoadjuvant and adjuvant immunotherapy in renal cell carcinoma. World J Urol.

[5] Bellocco R., Pasquali E., Rota M., Bagnardi V., Tramacere I., Scotti L. (2012). Alcohol drinking and risk of renal cell carcinoma: results of a meta-analysis. Ann Oncol.

[6] Baldewijns M.M., van Vlodrop I.J., Schouten L.J., Soetekouw P.M., de Bruine A.P., van Engeland M. (2008). Genetics and epigenetics of renal cell cancer. Biochim Biophys Acta.

[7] Li F., Aljahdali I.A.M., Zhang R., Nastiuk K.L., Krolewski J.J., Ling X. (2021). Kidney cancer biomarkers and targets for therapeutics: survivin (BIRC5), XIAP, MCL-1, HIF1alpha, HIF2alpha, NRF2, MDM2, MDM4, p53, KRAS and AKT in renal cell carcinoma. J Exp Clin Cancer Res.

[8] Kim H., Shim B.Y., Lee S.J., Lee J.Y., Lee H.J., Kim I.H. (2021). Loss of Von Hippel-Lindau (VHL) Tumor Suppressor Gene Function: VHL-HIF Pathway and Advances in Treatments for Metastatic Renal Cell Carcinoma (RCC). Int J Mol Sci.

[9] Latif F., Tory K., Gnarra J., Yao M., Duh F.M., Orcutt M.L. (1993). Identification of the von Hippel-Lindau disease tumor suppressor gene. Science.

[10] Gossage L., Eisen T., Maher ER. (2015). VHL, the story of a tumour suppressor gene. Nat Rev Cancer.

[11] Ricketts C.J., De Cubas A.A., Fan H., Smith C.C., Lang M., Reznik E. (2018). The cancer genome atlas comprehensive molecular characterization of renal cell carcinoma. Cell Rep.

[12] Gerlinger M., Rowan A.J., Horswell S., Math M., Larkin J., Endesfelder D. (2012). Intratumor heterogeneity and branched evolution revealed by multiregion sequencing. N Engl J Med.

[13] Cramer T., Yamanishi Y., Clausen B.E., Forster I., Pawlinski R., Mackman N. (2003). HIF-1alpha is essential for myeloid cell-mediated inflammation. Cell.

[14] Jonasch E., Donskov F., Iliopoulos O., Rathmell W.K., Narayan V.K., Maughan B.L. (2021). Belzutifan for Renal Cell Carcinoma in von Hippel-Lindau Disease. N Engl J Med.

[15] Mole D.R., Blancher C., Copley R.R., Pollard P.J., Gleadle J.M., Ragoussis J. (2009). Genome-wide association of hypoxia-inducible factor (HIF)-1alpha and HIF-2alpha DNA binding with expression profiling of hypoxia-inducible transcripts. J Biol Chem.

[16] Wiesener M.S., Jurgensen J.S., Rosenberger C., Scholze C.K., Horstrup J.H., Warnecke C. (2003). Widespread hypoxia-inducible expression of HIF-2alpha in distinct cell populations of different organs. FASEB J.

[17] Courtney K.D., Infante J.R., Lam E.T., Figlin R.A., Rini B.I., Brugarolas J. (2018). Phase I dose-escalation trial of PT2385, a first-in-class hypoxia-inducible factor-2alpha antagonist in patients with previously treated advanced clear cell renal cell carcinoma. J Clin Oncol.

[18] Takamori H., Yamasaki T., Kitadai R., Minamishima Y.A., Nakamura E. (2023). Development of drugs targeting hypoxia-inducible factor against tumor cells with VHL mutation: Story of 127 years. Cancer Sci.

[19] Kallemeijn M.J., Kavelaars F.G., van der Klift M.Y., Wolvers-Tettero I.L.M., Valk P.J.M., van Dongen J.J.M. (2018). Next-generation sequencing analysis of the Human TCRgammadelta+ T-cell repertoire reveals shifts in vgamma- and vdelta-usage in memory populations upon. Aging Front Immunol.

[20] Cheng C., Wang B., Gao L., Liu J., Chen X., Huang H. (2018). Next generation sequencing reveals changes of the gammadelta T cell receptor repertoires in patients with pulmonary tuberculosis. Sci Rep.

[21] Kolinsky M.P., Niederhoffer K.Y., Kwan E.M., Hotte S.J., Hamilou Z., Yip S.M. (2021). Considerations on the identification and management of metastatic prostate cancer patients with DNA repair gene alterations in the Canadian context. Can Urol Assoc J.

[22] Casto A.M., Fredricks D.N., Hill JA. (2021). Diagnosis of infectious diseases in immunocompromised hosts using metagenomic next generation sequencing-based diagnostics. Blood Rev.

[23] Crumley S.M., Divatia M., Truong L., Shen S., Ayala A.G., Ro JY. (2013). Renal cell carcinoma: Evolving and emerging subtypes. World J Clin Cases.

[24] Amin M.B., Edge S.B., Greene FL. (2017).

[25] Jin Y., Chen D.L., Wang F., Yang C.P., Chen X.X., You J.Q. (2020). The predicting role of circulating tumor DNA landscape in gastric cancer patients treated with immune checkpoint inhibitors. Mol Cancer.

[26] Zhou Q., Tao F., Qiu L., Chen H., Bao H., Wu X. (2022). Somatic alteration characteristics of early-onset gastric cancer. J Oncol.

[27] Wang K., Li M., Hakonarson H. (2010). ANNOVAR: functional annotation of genetic variants from high-throughput sequencing data. Nucleic Acids Res.

[28] Ng P.C., Henikoff S. (2003). SIFT: Predicting amino acid changes that affect protein function. Nucleic Acids Res.

[29] Adzhubei I., Jordan D.M., Sunyaev S.R. (2013). Predicting functional effect of human missense mutations using PolyPhen-2. Curr Protoc Hum Genet.

[30] Tomczak K., Czerwinska P., Wiznerowicz M. (2015). The cancer genome atlas (TCGA): an immeasurable source of knowledge. Contemp Oncol (Pozn).

[31] Sato Y., Yoshizato T., Shiraishi Y., Maekawa S., Okuno Y., Kamura T. (2013). Integrated molecular analysis of clear-cell renal cell carcinoma. Nat Genet.

[32] Mitchell T.J., Turajlic S., Rowan A., Nicol D., Farmery J.H.R., O'Brien T. (2018). Timing the landmark events in the evolution of clear cell renal cell cancer: TRACERx renal. Cell.

[33] de Cubas A.A., Rathmell W.K. (2018). Epigenetic modifiers: activities in renal cell carcinoma. Nat Rev Urol.

[34] (2013). Cancer genome atlas research N. Comprehensive molecular characterization of clear cell renal cell carcinoma. Nature.

[35] Hogner A., Krause H., Jandrig B., Kasim M., Fuller T.F., Schostak M. (2018). PBRM1 and VHL expression correlate in human clear cell renal cell carcinoma with differential association with patient's overall survival. Urol Oncol.

[36] da Costa W.H., da Cunha I.W., Fares A.F., Bezerra S.M., Shultz L., Clavijo D.A. (2018). Prognostic impact of concomitant loss of PBRM1 and BAP1 protein expression in early stages of clear cell renal cell carcinoma. Urol Oncol.

[37] da Costa W.H., Rezende M., Carneiro F.C., Rocha R.M., da Cunha I.W., Carraro D.M. (2014). Polybromo-1 (PBRM1), a SWI/SNF complex subunit is a prognostic marker in clear cell renal cell carcinoma. BJU Int.

[38] Jiang W., Dulaimi E., Devarajan K., Parsons T., Wang Q., O'Neill R. (2017). Intratumoral heterogeneity analysis reveals hidden associations between protein expression losses and patient survival in clear cell renal cell carcinoma. Oncotarget.

[39] Nam S.J., Lee C., Park J.H., Moon KC. (2015). Decreased PBRM1 expression predicts unfavorable prognosis in patients with clear cell renal cell carcinoma. Urol Oncol.

[40] Joseph R.W., Kapur P., Serie D.J., Eckel-Passow J.E., Parasramka M., Ho T. (2014). Loss of BAP1 protein expression is an independent marker of poor prognosis in patients with low-risk clear cell renal cell carcinoma. Cancer.

[41] Thiesen H.J., Steinbeck F., Maruschke M., Koczan D., Ziems B., Hakenberg OW. (2017). Stratification of clear cell renal cell carcinoma (ccRCC) genomes by gene-directed copy number alteration (CNA) analysis. PLoS One.

[42] Cossu-Rocca P., Muroni M.R., Sanges F., Sotgiu G., Asunis A., Tanca L. (2016). EGFR kinase-dependent and kinase-independent roles in clear cell renal cell carcinoma. Am J Cancer Res.

